# Incarcerated paraesophageal hernia complicated by pancreatic damage and unusual comorbidity: Two retrospective case series

**DOI:** 10.1016/j.ijscr.2018.11.064

**Published:** 2018-11-30

**Authors:** H.M. Haug, E. Johnson, T. Mala, D.T. Førland, T.T. Søvik, H.O. Johannessen

**Affiliations:** a Department of Pediatric and Gastrointestinal Surgery, Oslo University Hospital, Ullevål, P.O. box 4950, 0424 Oslo, Norway; b Institute of Clinical Medicine, University of Oslo, Kirkeveien 166, 0450 Oslo, Norway

**Keywords:** PEH, paraesophageal hernia, Case series, Incarcerated paraesophageal hernia, Ischemia, Pancreatic fistula

## Abstract

Introduction: About 1% of paraesophageal hernias (PEH) require emergency surgery due to obstruction or gangrene. We present two complicated cases of incarcerated PEH.

Presentation of cases: A patient aged 18 with trisomy 21 was admitted after four days of vomiting and epigastric pain. CT scan revealed a large PEH. The stomach was massively dilated with compression of adjacent viscera and the celiac trunk. The stomach was repositioned laparoscopically and deflated by endoscopy in an attempt to avoid resection. During second look laparoscopy a gastrectomy was necessary. The patient was reoperated for intestinal obstruction, and treated for dehiscence of the esophagojejunostomy and a pancreatic fistula. A patient aged 65 with hereditary spastic paresis had two days history of emesis and epigastric pain. Upon arrival he was hemodynamically unstable and a CT scan revealed perforation of the herniated stomach. A subtotal gastrectomy without reconstruction was performed with vacuum closure of the abdomen. Later a gastrectomy was completed with a Roux-en-Y reconstruction. Except from reoperation for wound dehiscence after 14 days, the recovery was uneventful.

Discussion: Trisomy 21 and hereditary spastic paresis may increase the risk of developing PEH. Challenges in regard to symptom evaluation may delay diagnosis. The pressure of the dilated stomach can give rise to ischemic and mechanical damage from compression of major blood vessels and organs. Urgent diagnosis and gastric deflation is required.

Conclusions: In patients with known PEH or with comorbidity that may increase the risk of PEH, this diagnosis should be considered early on.

## Highlights

• We present two cases of paraesophageal hernia that both needed total gastrectomy due to gangrene.
• Both patients had clinical relevant comorbidities, respectively trisomy 21 and hereditary spastic paresis.
• Due to compression from the dilated stomach one of the patients developed ischemia of the pancreas with leakage of peptidases which in turn caused anastomotic dehisence and intraabdominal abscess.
• The pancreatic damage and anastomotic leakage was treated conservatively with repeated stenting and percutaeous drainage.
• Immediate diagnosis and treatment for incarcerated paraesophageal hernias are vital to reduce morbidity and mortality.

## 1 Introduction

The prevalence of paraesophageal hernia (PEH) is unknown as many are asymptomatic. Typical manifestations include gastroesophageal reflux, food retention, aspiration pneumonia, and epigastric pain. Pooled estimates reveal that 9.2% of patients acutely admitted with PEH obstruction may have incarceration with gangrene [1]. The annual probability of developing symptoms requiring acute surgery for patients with PEH was 1.2% [2]. The overall 30 and 90-day mortality in emergency cases in a cohort of 12,441 patients was 7% and 11.5%, respectively [1]. In case of obstruction emergent intervention is required. In some patients early diagnosis, however, may be challenging and adequate treatment could be delayed. We report two patients, one with trisomy 21 and another with hereditary spastic paresis who were treated for incarcerated PEH at our Institute. The work has been reported in line with the SCARE criteria and the PROCESS CHECKLIST [3,4]

## 2 Case presentations

**Fig. 1:**
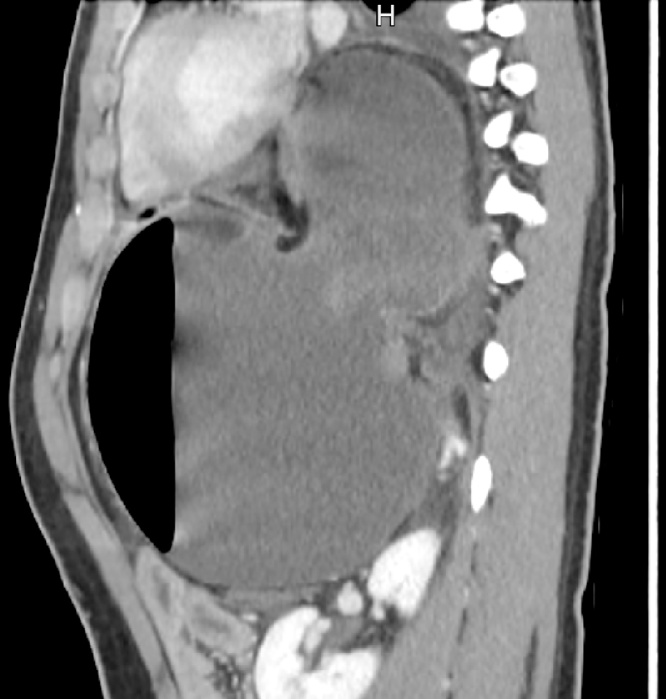
Patient 1, prior to surgery. Herniation of the distended stomach into the thoracic cavity. The dilatation above and below diaphragm is shown.

**Fig. 2:**
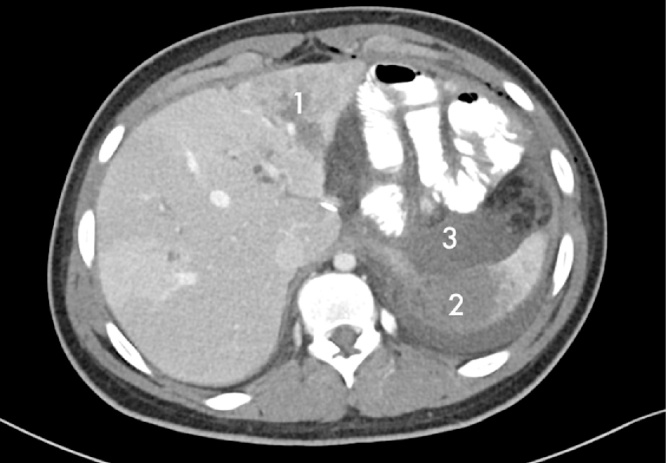
Patient 1. CT on second postop. day. Infarction in the left liver lobe (1) and the spleen (2) with neighbouring fluid collection (3).

**Fig. 3:**
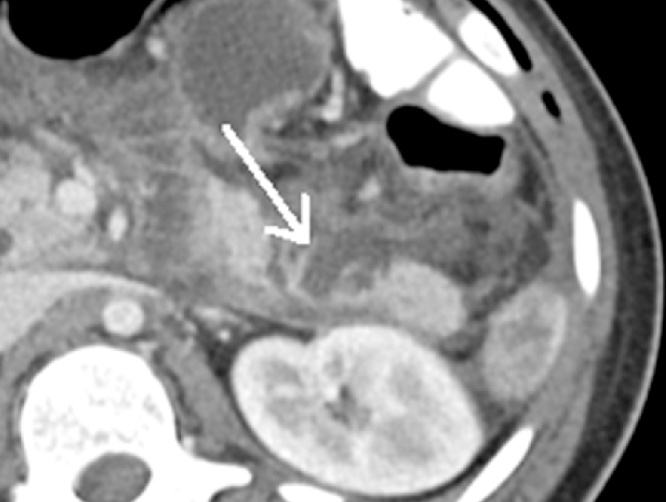
Patient 1. CT on second postop day. Lack of perfusion (arrow) of a distal segment of the pancreatic body.

**Fig. 4:**
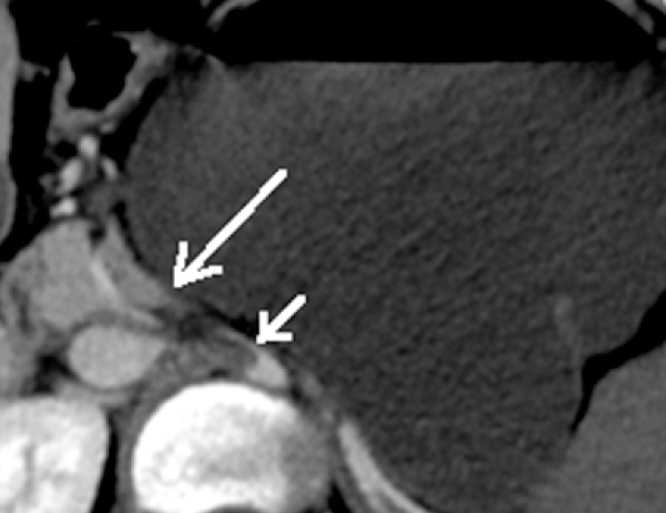
Patient 1. Preoperative CT. Compression of the pancreas (large arrow) and the coeliac trunk (arrow) from a massively dilated stomach.

**Fig. 5:**
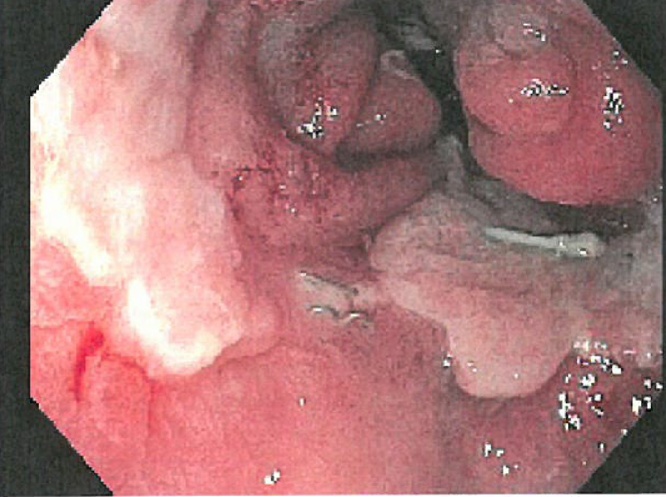
Patient 1. After final stent removal the anastomosis was intact and open.

**Fig. 6:**
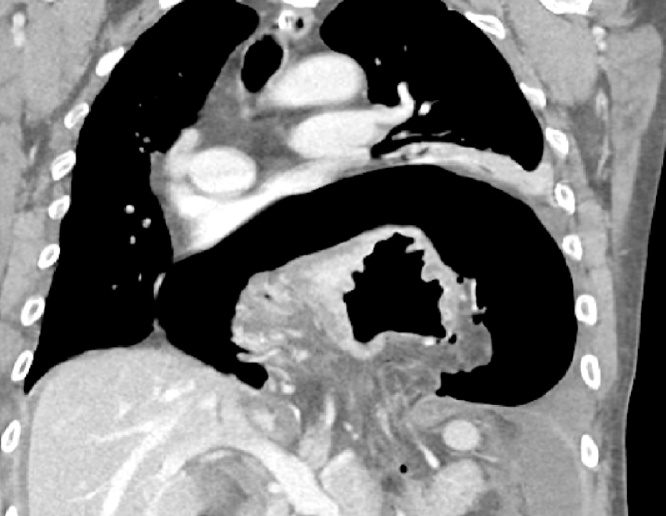
Patient 2 prior to surgery. Free air around the perforated stomach encircled by the peritoneum.

Patient 1. A patient aged 18 with trisomy 21 with impacted verbal language and symptomatic recurrence after Nissen’s fundoplication in 2007, was admitted to the local hospital for vomiting and abdominal pain for four days. CT scan revealed that proximal 2/3 of the stomach was herniated into the left hemithorax (Fig. 1). The outlet of the abdominal part of the stomach was obstructed. A nasogastric tube was attempted without success and the patient was transferred to our Institute. Immediate gastroscopy was attempted, but advancement to the intra-abdominal part of the stomach was unsuccessful. At acute surgery the ischemic stomach with hemorrhagic mucosa was repositioned into the abdomen and the stomach was drained by endoscopy. Extent of ischemia and viability of the affected stomach was uncertain and a planned second stage laparoscopy was opted for two days later. The following day the patient was septic. Because of ischemic damage to large parts of the stomach a laparoscopic gastrectomy with Roux-en-Y reconstruction was performed using a 25 mm Medtronic DST series™ EEA™ ORVIL™ device to construct a circular stapled anastomosis. The crurae were approximated by non-absorbable sutures and enforced by a 7 × 10 cm horseshoe shaped BioDesign® mesh before placement of a percutaneous jejunal catheter for nutrition. A repeat CT scan postoperatively demonstrated abdominal fluid and infarctions of the upper part of the spleen and peripherally in the left liver lobe (Fig. 2). There was also lack of perfusion of a segment of the pancreatic body (Fig. 3). The preoperative CT scan was reinvestigated and revealed extensive compression by the extended and undrained stomach affecting the pancreas and the coeliac trunk (Fig. 4). A pigtail catheter drained the accumulated fluid that contained amylase. Due to dilatation of the biliopancreatic limb of the Roux-en -Y reconstruction a laparotomy was performed four days after initial surgery demonstrating herniation of the biliopancreatic limb through Petersen’s space. The herniation was reduced and the mesenteric defect was sutured. A drain was positioned towards the pancreas. Octreotide and antibiotics were administered and the patient gradually improved. At day 17 he had fever and vomiting and a defect in the esophagojejunostomy was confirmed by endoscopy. A sump tube placed through the defect was held on continuous suction for four days before insertion of a partially covered Endoflex® stent. Following endoscopic evaluation of the defect after two weeks the treatment was continued with an identical stent. The patient started to eat and was transferred to the local hospital at day 38 and discharged home at day 54. The patient was readmitted to our Institute at day 58. A CT scan demonstrated gastrojejunal leakage, a mediastinal fluid collection and an abscess adjacent to the pancreatic tail. Percutaneous abscessography and drainage demonstrated passage of contrast through the anastomosis no longer covered by the distally migrated stent. There was near total anastomotic dehiscence. The stent was removed and the following day a wire was successfully advanced into jejunum and a third stent positioned using an Egis e-PTFE stent. A control CT scan on day 63 showed increasing intraabdominal fluid collections because of still active pancreatic fistulation. CT-guided drainage was performed at the pancreatic tail, left pleura and flank, epigastrium and suprapubic, respectively. He gradually improved and the third stent was removed at day 85. A forth stent (Endoflex) was necessary until day 103 for complete healing of the anastomosis (Fig. 5). He was transferred to the local hospital at day 104. At discharge (day 134) he was allowed intake of food, but still received nutritional supplements through the jejunal catheter. Patient 2. A man aged 65, with hereditary spastic paresis was admitted to the local hospital after one day with upper abdominal pain and emesis. A CT scan the following day showed a hiatal hernia with about two thirds of a dilated stomach dislocated into the thoracic cavity. After placement of a nasogastric tube that returned some blood, the patient was transferred to a central hospital where the stomach was deflated by endoscopy. However, due to clinical deterioration with development of septicaemia the patient was transferred to our institute. Repeat CT showed free air around the herniated stomach and fluid in the abdomen (Fig. 6). At laparotomy a large perforation was detected in a necrotic area on the posterior wall of the gastric body. A subtotal gastrectomy was performed by removing the gastric antrum and most of the gastric body. Because of septicemia and hemodynamic instability, no anastomoses were made and vacuum closure of the abdomen was performed. Two days later a second-look operation was conducted with abdominal lavage followed by resection of the hernia-sac and a stapled Roux-en-Y gastrectomy. The hiatus was too wide for adequate crural approximation and a mesh was not used because of contamination. Besides episodes of atrial fibrillation the postoperative course was uncomplicated until day 13 when the patient had transient subileus because of a wound dehiscence that was closed. The patient was allowed to drink clear fluids and the nasoenteral tube was removed on day 25 when oral feeding gradually started. The patient was discharged from the local hospital for rehabilitation at day 58.

## 3 Discussion

The pathophysiology of PEH is complex. Contributory factors are increased intra-abdominal pressure, hiatal widening because of congenital or acquired changes of neighbouring muscle and connective tissue and esophageal shortening from fibrosis and excessive vagal nerve stimulation [5,6]. Trisomy 21 is not a syndrome typically related to PEH, but associated with altered motility of the GI-tract with increased incidence of gastroesophageal reflux, achalasia and Hirschsprung`s disease [7,8]. A Nissen`s fundoplication for gastroesophageal reflux at age 9 could have been a contributing risk factor for development of PEH [9]. The remarkably well-developed abdominal musculature due to the spasticity in patient 2 suggested a chronically increased intra-abdominal pressure as a contributing factor to development of PEH. Patients with incarcerated PEH may experience delayed diagnosis due to unspecific symptoms that mimic other conditions such as cardiovascular disease [10]. Inability to communicate may also delay diagnosis as was the case in the patient with trisomy who was initially was thought to have gastroenteritis. The stomach is most efficiently emptied by endoscopy and kept deflated by placement of a nasogastric tube. This may reduce the risk for gastric ischemia and pneumonia from gastric aspiration. In patient 1 endoscopy was unsuccessful as it was not possible to advance into the intraabdominal part of the stomach and emergency surgery was inevitable. Based on preoperative imaging and the delay in diagnosis and stomach deflation we suspect that ischemic damage with partial disruption of a segment the pancreatic body was caused by preoperative compression by the massively dilated stomach (Fig. 4). The decision to percutaneously drain the pancreatic fistula seemed justified as it was a safe strategy although with a healing time of about 3,5 months. Insertion of a pancreatic stent via papilla Vateri could have shortened healing of the fistula. However, ERCP was considered too risky because of the insufficient anastomosis and the Roux-en-Y reconstruction. Especially in this patient an alternative strategy with pancreatic resection was considered a procedure with a higher probability for major complications. The pancreatic fistulation probably contributed to the leak and delayed healing at the gastrojejunostomy. To our knowledge the combination of PEH with gastric necrosis complicated with pancreatic ischemia and fistula, has hitherto not been reported. The patient with hereditary spastic paresis had a PEH where gastric perforation necessitated a gastrectomy. A potential increased abdominal pressure due do hereditary condition probably both contributed to herniation and wound dehiscence, in an otherwise satisfactorily recovery.

## 4 Conclusions

Patients with Trisomy 21 and hereditary spastic paresis may have increased risk of incarcerated PEH that is a condition with high risk of morbidity and mortality. Early diagnosis and gastric emptying are the main preventive measures in order to reduce complications and need for radical surgery in general, and not least for these patients.

## Conflicts of interest

None of the authors have any conflicts of interest in regards of writing this article.

## Funding

This research did not receive any specific grant from funding agencies in the public, commercial, or not-for-profit sectors.

## Ethical approval

This case series in not within the mandate for the regional ethical committee.

## Consent

Written informed consent was obtained from the patients for publication of this case series and accompanying images. A copy of the written consent is available for review by the Editor-in-Chief of this journal on request.

## Author contribution

Helene M. Haug has written the case series and included relevant figure legends highlighting and picturing two separate cases. Egil Johnson has evaluated the case series and contributed with close co-operation in outlining, writing and including of relevant information. Tom Mala, Torgeir Thorson Søvik, Dag Tidemann Førland and Hans-Olaf Johannessen have contributed to the surgery of these patients and critical revision of the manuscript.

## Registration of research studies

researchregistry4514.

## Guarantor

Egil Johnson.

## Provenance and peer review

Not commissioned, externally peer-reviewed
